# Heterogeneity of non-suicidal self-injury behavior in adolescents with depression: latent class analysis

**DOI:** 10.1186/s12888-023-04808-7

**Published:** 2023-05-01

**Authors:** He He, Lan Hong, Wei Jin, Yao Xu, Wei Kang, Jie Liu, Jingyao Song, Tiansheng Zheng, Hong Chen, Ke Zhao

**Affiliations:** 1 School of Mental Health, Wenzhou Medical University, Wenzhou, 325035 China; 2 The Third Hospital of QuZhou, Quzhou, 324003 China; 3 The Affiliated Kangning Hospital of Wenzhou Medical University, Wenzhou, China; 4 Department of Psychiatry, First Affiliated Hospital of Wenzhou Medical University, Wenzhou, China; 5 Lishui Second People’s Hospital Affiliated to Wenzhou Medical University, Lishui, China; 6 The Affiliated Kangning Hospital of Wenzhou Medical University Zhejiang Provincial Clinical Research Center for Mental Disorder, Wenzhou, China

**Keywords:** Non-suicidal self-injury, Latent class analysis, Depression, Adolescent, Suicide

## Abstract

**Background:**

Non-suicidal self-injury (NSSI) by adolescent patients with depression has become a serious public health problem. This cross-sectional study aims to identify subgroups of adolescents based on NSSI and explore the factors related to these subgroups.

**Methods:**

The study recruited 326 in- and out-patient adolescents (263 girls and 63 boys) aged 12 to 18 years (mean = 14.7, SD = 1.6) who had self-injured in the past year. Latent class indicators included 12 NSSI variables, as well as suicidal ideation. Logistic regression examined associations between identified classes and related factors.

**Results:**

In this study, two distinct subgroups were identified: a “high suicidal ideation NSSI group” (n = 129, 39.6%) and a “low suicidal ideation NSSI group” (n = 197, 60.4%). Depression (OR = 1.10; 95% CI, 1.05–1.16), female (OR = 2.01; 95% CI, 1.09–3.69), left-behind experience (OR = 2.08; 95% CI, 1.17–3.71), single-parent family (OR = 1.84; 95% CI, 1.11–3.04) and peer victimization (OR = 1.04; 95% CI, 1.02–1.05) increases the probability of belonging to the “high suicidal ideation NSSI group”. A high level of perceived social support (OR = 0.99; 95% CI, 0.97–0.99) was a protective factor towards NSSI.

**Conclusions:**

This study identifies two subgroups of NSSI and the factors associated with each subgroup. The early identification of high-risk groups for major NSSI in adolescents diagnosed with depression is possible due to the identification of correlating factors. Different treatment plans can be developed for different subtypes of NSSI to improve the effectiveness of prevention and intervention, promoting the healthy physical and mental development of adolescents with depression.

**Supplementary Information:**

The online version contains supplementary material available at 10.1186/s12888-023-04808-7.

## Introduction

Non-suicidal self-injury (NSSI) is defined as an act of intentionally causing harm to own self without suicidal intent [1]. NSSI usually first occurs in early-to-middle adolescence. A systematic review of longitudinal studies on NSSI showed that prevalence rates of NSSI peak around mid-adolescence (around 15–16 years) [2]. A meta-analysis found that the prevalence of NSSI in children and adolescents is 19.5% worldwide [3], however, in China, the estimated prevalence in middle school students (aged 13–18 years) is 27.4% [4].

NSSI is associated with psychopathology, and psychiatric disorders were present in more than 80% of self-injuring patients presenting to general hospitals, with depression, anxiety, and alcohol abuse disorders being the most common [5]. Studies have shown that depressed mood and NSSI often co-occur [6], with depression being one of the strongest correlates of NSSI [2]. As such, NSSI engagement is often seen with a diagnosis of major depression [7]. Adolescents reporting depression and NSSI have poorer interpersonal functioning and pain threshold, and may have higher rates of suicidal ideation than adolescents without NSSI [8–10]. The presence of comorbidity in adolescents may lead to a longer duration of depression and severe depressive symptoms [11]. As the comorbidity of depression and NSSI may affect the clinical prognosis, more attention should be focused on this population. However, most NSSI does not receive adequate psychosocial assessment or care from health professionals, which increases the risk of suicide attempts (SA) and exacerbates related mental health concerns [12].

NSSI has also been associated with SA [13]. In clinical samples, up to 70% of NSSI adolescents report a history of SA [7]. NSSI history is more predictive of future suicide than SA history, and individuals with NSSI history are 30 times more likely to complete suicide than general population [14]. A mortality follow-up study was conducted on 11,583 patients, and the results showed that the risk in the first year of follow-up was 66 times the annual risk of suicide in the general population. The risk after five years was 1.7%, at ten years 2.4%, and at 15 years 3.0% [15]. Such an extended association suggests that NSSI is the most critical risk factor for suicide in the future.

In addition, alexithymia and peer victimization are also closely associated with NSSI in adolescents. Bullying has been linked to internalizing symptoms like depression [16] and is a risk factor for the development of recurrent NSSI [17]. In line with the experiential avoidance modes of NSSI, NSSI is often used by victimized youth to avoid or relieve negative emotions caused by bullying [18]. Furthermore, alexithymia may be a significant factor in the emergence of NSSI [19]. Alexithymia is a personality factor defined as an impairment in identifying and describing emotion [19]. Tang et al. [20] found that alexithymia can positively predicted NSSI, and depression may play a mediating role between alexithymia and NSSI. Alexithymic individuals frequently use more suppression techniques to control their emotions [21], which can lead to intensely negative feelings and an increased propensity for NSSI [22]. In terms of the protective factors, psychological resilience and social support were also associated with NSSI in adolescents with depression. Jessica’s [23] research found that higher psychological resilience among teenagers who have endured bad experiences like bullying can lessen the risk of NSSI by reducing depressed symptoms. Low level of social support is also linked to the start of NSSI and negative emotion [24]. As a result, these aspects should be taken into account when seeking to define psychosocial factors connected to NSSI typologies.

Not all teenagers with depression engage in NSSI, with some research suggesting that NSSI individuals may not form a homogeneous group. A high degree of disease heterogeneity makes diagnosis and individualized treatment more difficult. Most of the current clinical staging is based on the academic consensus of symptomatology, which relies on clinicians’ perceptions rather than on adequate data analysis. The basis of disease staging lacks evidence-based medical evidence and may not reflect the actual disease situation [25]. So by identifying clusters of symptoms within patients who present with NSSI, diagnoses and interventions can be tailored to each patient.

Some research has suggested the classification of NSSI to help explain the heterogeneity in the patterns of NSSI. Researchers and clinicians used modifiers such as mild, moderate, and severe when classifying NSSI, mimicking previous psychiatric disorder classifications (major depressive episodes) [26]. Many previous studies have identified subgroups based on life-time frequency and defining features of self-injury [27], the nature of NSSI and gender [28], and co-occurrence with suicidal ideation and attempts [29]. Furthermore, some empirical studies attempted to classify NSSI using statistical methods. Factor analysis has been used to categorize eleven NSSI behaviors into two subtypes: moderate/severe NSSI and minor NSSI [30]. In addition, there are classification methods that use cluster analysis and group-based trajectory modeling [31, 32]. Given conventional analysis for NSSI characteristics, descriptive variables did not disclose the complexity of NSSI patterns, leading to overly general conclusions [33]. Many studies have begun to utilize more robust statistical methods for exploring the heterogeneity in NSSI patterns.

Latent class analysis (LCA) is a typical unsupervised machine learning method [34]. LCA is more statistically principled than the standard non-hierarchical and hierarchical clustering techniques. The statistical inference is built from a probability model assumed to hold in the data [34]. In contrast to variable-centered modeling approaches, LCA can identify “hidden” subgroups of individuals with a distinct pattern of abuse subtypes that cannot be directly observed. Information on risk characteristics associated with latent class membership can more readily inform personalized clinical practice [35]. Although several latent variable models have been successfully formulated to identify the heterogeneity in NSSI patterns, most studies focused on community adolescent populations [31]. The first LCA analysis identified four subgroups of NSSI, including the experimental NSSI group, mild NSSI group, multiple functions/anxious group, and automatic functions/suicidal group, which differed on key clinical variables [27]. Hamza conducted an LCA using several characteristics of NSSI and suicidal behaviors as class indicators. Three subgroups of NSSI were identified, infrequent NSSI/not high risk for suicidal behavior group, a frequent NSSI/not high risk for suicidal behavior group, and a frequent NSSI/high risk for suicidal behavior group [36]. A recent study analysis yielded four subgroups of NSSI, mild/experimental NSSI, moderate NSSI, moderate multiple functions NSSI and severe NSSI group [37]. These studies confirm the presence of numerous sub-groups of NSSI, suggesting that the different subclasses need to be further explored and differentiated.

Many previous studies have focused on classifying NSSI in community samples using LCA, with few studies conducted in China to validate the heterogeneity of NSSI in clinical samples. In contrast to non-clinical samples, patients in clinical settings suffering from more mental health problems related to NSSI, such as anxiety, depression and alexithymia [38]. The field of research on NSSI has focused on Caucasian Western patients [39]. The prevalence of NSSI varies across studies due to factors, such as different definitions and assessment tools for NSSI, and cultural differences between countries [40]. China has a substantially different cultural background from Western nations, emphasizing social relationships and moral norms, while adolescence is a time when self-awareness is gradually emerging. As a result, the conflict between an individual’s internal wants and their external environment may be more pronounced and intense in Chinese adolescents [41]. From this perspective, NSSI as a form of coping may be more prevalent among Chinese adolescents than in the West [42, 43]. Recently, the prevalence of NSSI among Chinese adolescents has been increasing yearly and is gradually receiving attention from families, schools, and society. Therefore, early identification and intervention for NSSI are imminent [44]. NSSI among adolescents is a common clinical problem, but the severity and prognosis of NSSI vary among adolescents and may be related to many factors, such as gender, age, single-parent family, left-behind experiences (this refers to adolescents whose parents or one of their parents used to be migrants, and could not live with parents in their areas of origin during childhood), bullying, and alexithymia. The frequency of NSSI modalities is correlated with NSSI outcomes and therefore were included in this study.

This study aims to describe the heterogeneous sub-groups of NSSI in a clinical sample of Chinese adolescents using LCA, while examining NSSI’s behavioral characteristics and social psychological factors among various NSSI classes. Previous studies have found that individuals who use multiple NSSI methods have a higher risk of suicidal behavior than those who use fewer NSSI methods [7]. NSSI method and suicidal ideation have also been used as latent variables of NSSI and utilized in previous LCA studies [36]. This study aims to describe the heterogeneous sub-groups of NSSI in a clinical sample of Chinese adolescents using LCA based on the different types of NSSI behavior and suicidal variables, and examines NSSI’s behavioral characteristics and social psychological factors among various NSSI classes.

## Method

### Participants

Data were derived from a cross-sectional study of NSSI among adolescents in the Department of Psychiatry, First Affiliated Hospital of Wenzhou Medical University and The Affiliated Kangning Hospital of Wenzhou Medical University. All participants were assessed and screened by a psychiatrist with an attending title or higher according to the Structured Clinical Interview for DSM-V, Patient version (SCID-I/P) to clarify the diagnosis and determine enrollment. All participants met the diagnostic criteria of moderate to severe depressive episodes in adolescents or bipolar disorder with a current depressive episode. Informed consent was obtained from all participants and/or their legal guardians.

The inclusion criteria for the study were: (1) 12–18 years old; (2) years of education ≥ 5 years; (3) depressive disorder or bipolar disorder and is currently in a depressive episode; (4) had NSSI within the past year; (5) voluntarily signed informed consent.

The exclusion criteria were: (1) suffering from any severe physical, infectious, or immune system diseases; (2) history of severe mental disorders, such as schizophrenia or mental retardation; (3) patients with traumatic brain injury, epilepsy or other known severe neurological or brain disorders; (4) withdrawal of informed consent or failure to complete the scale.

**Fig. 1:**
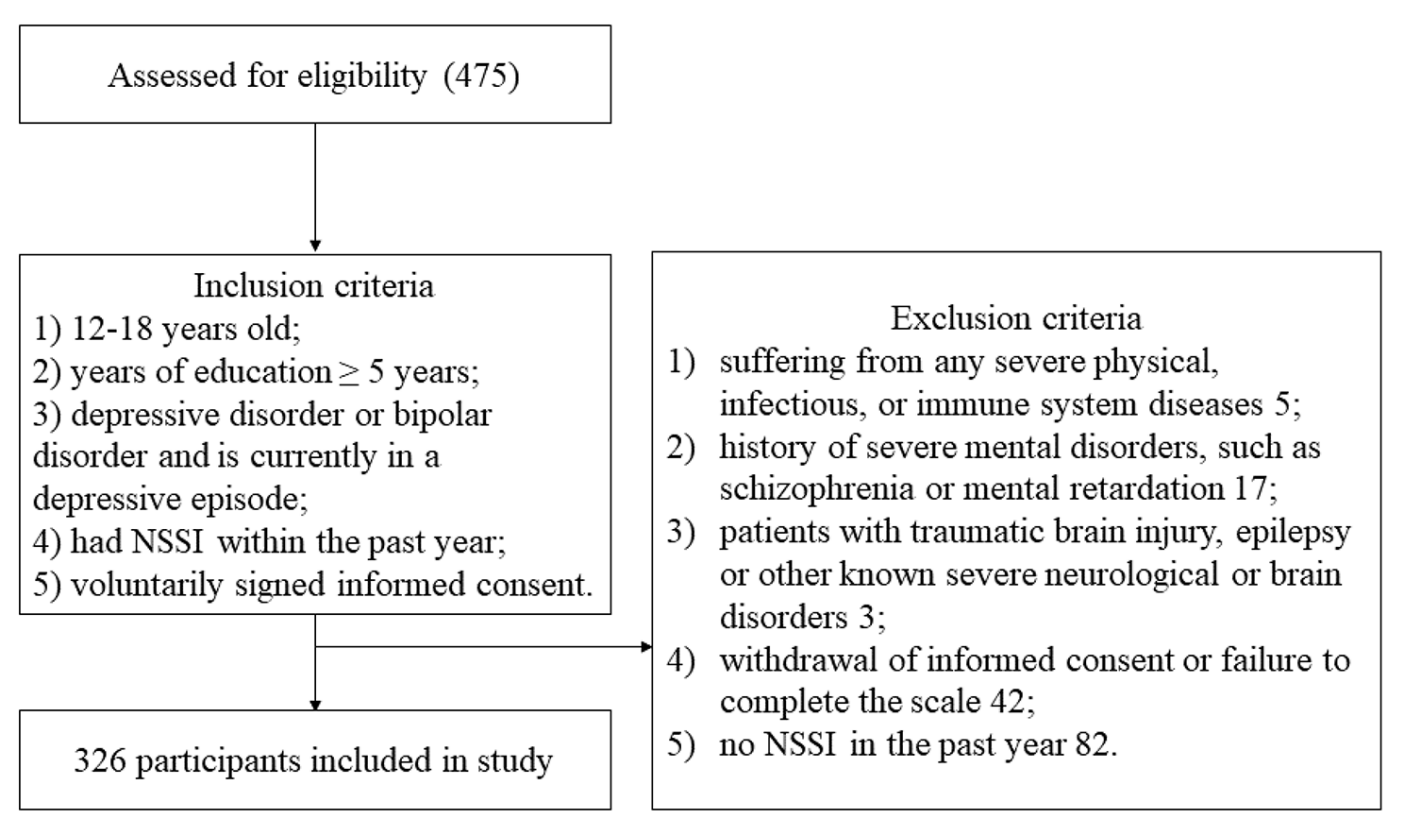
Sample flow chart

Convenience sampling was used to select 475 adolescents from out-patient and in-patient settings. A total of 149 adolescents did not meet the inclusion criteria and were excluded. Analyses were conducted on 326 adolescents aged 12 to 18 (mean = 14.7, SD = 1.6), including 263 females and 63 males. Detailed information regarding participation was presented in Fig. 1.

### Procedure

First, all participants were evaluated by one attending psychiatrist or higher and met the diagnosis of a depressive episode. The testers were all psychology graduate students and were trained to ensure they were familiar with the study process before the study formally began. The questionnaires were completed anonymously and were numerically coded, the testers and data analyzers didn’t know the participants’ names. All tests are carried out in a quiet room or ward using a tablet computer and take approximately 30 min. Participants completed anonymous surveys administered in a group during weekday hours. All participants needed to complete the questionnaire independently and receive the score results at the end of the test. In the written debriefing statement, students who wish to discuss the issues raised in the questionnaire package in more detail will do so in person with the psychiatrist. Before the research was carried out, the study protocol was reviewed and approved by the Research Ethics Committee, Wenzhou Medical University.

## Measures

### Self‑report demographic survey

The participants filled out the social and demographic section of the survey: age, height, weight, location (city, rural), years of education (≤ 9 years, 9–12 years), Left-behind experience (Yes, No), only children (Yes, No), single-parent family (Yes, No), family history of mental disorders (Yes, No).

### Suicidal ideation and suicide attempt

A single item was used to assess participants’ SA and ideation. Participants were asked, “Have you attempted suicide/had suicidal ideation in the past 12 months?“. These one-item measures of SI and SAs have been used in previous studies [45, 46].

### Depression

The Patient Health Questionnaire-9 (PHQ-9) consists of 9 items on a 4-point Likert scale from 0 (none/seldom time) to 4 (most of the time/all of the time) to assess depression in adolescents. Studies have shown that the PHQ-9 can evaluate and compare the severity of depression across age and gender during adolescence [47]. The Cronbach’α was 0.90.

### Non-suicidal self-injury

The Functional Assessment of Self-Mutilation (FASM) is a self-report measure of self-injury methods, frequency, and functions [48]. Participants first indicated whether and how often they had engaged in 12 different methods of self-injury in the previous 12 months, with space provided for any methods not listed. To assess the functions of NSSI, participants were then asked how often they had engaged in NSSI for each of 15 different reasons (scored from 0 = never to 3 = often for each item), with space provided for any reasons not listed. Other aspects of the participants’ NSSI, such as the age of onset, length of consideration before each NSSI, degree of pain at the time of the NSSI and whether suicidal thoughts occurred at the time of the act, were also assessed. The FASM has been used in studies of normative [49] and psychiatric samples [50], which have yielded support for its psychometric properties. In addition, the scale has also been confirmed to have reliability among Chinese clinical adolescents [48].

### Resilience

The Connor Davidson Resilience Scale-10 (CD-RISC-10) was used to assess participants’ levels of resilience. The scale contains ten items, scored on a 5-point Likert scale from 1 (never) to 5 (always), and is widely used to assess mental toughness in different populations, including adolescents, older adults and psychiatric patients [51], with higher scores implying higher mental toughness, and the Cronbach’α was 0.90.

### Alexithymia

The Toronto Alexithymia Scale (TAS) is a 20-item self-reported instrument, with each item rated on a 5-point Likert scale ranging from 1 (strongly disagree) to 5 (strongly agree). Total scores range from 20 to 100, with higher scores indicating higher degrees of alexithymia. The psychometric properties of the TAS-20-C are satisfactory among Chinese adolescents [52]. The Cronbach’α was 0.79.

### Peer victimization

Peer victimization was measured was measured by the 16-item Multidimensional Peer Victimization Scale (MPVS) [53]. The scale assesses four dimensions: physical victimization, verbal victimization, social manipulation, and attacks on property. The scale has good reliability and validity in the younger age group. The Cronbach’s α was 0.93.

### Perceived social support

The Multidimensional Scale of Perceived Social Support (MSPSS) measures the perceived adequacy of social support from three domains: family, friends, and significant others [54]. The scale comprises 12 items. Each item is a 7-point Likert scale, ranging from 1 (strongly disagree) to 7 (strongly agree). The higher the score, the more perceived social support is in place. The Cronbach’α was 0.92.

### Statistical analyses

Firstly, 326 adolescents with NSSI were selected for LCA using Mplus version 8.0. Latent class indicators included 12 NSSI variables (0: no, 1: yes), as well as the suicidal variables (e.g., suicidal ideation, 0: no, 1: yes). In LCA, models are compared to determine the optimal number of classes (i.e., class enumeration), beginning with evaluating the fit of a one-class model and incrementally adding latent classes until the best class solution has been satisfied [55].

Model selection considers several fit indices, including information criteria and likelihood ratios. The evaluation indicators of the degree of fit of LCA are the Akaike Information Criterion (AIC) [56], Bayesian Information Criterion (BIC) [57], and sample-size adjusted BIC (aBIC) [58]. These are relative metrics where lower values of BIC, AIC, and aBIC are better. The second is the entropy value, with a maximum value of 1, and higher values are preferred [59]. An entropy value greater than 0.8 indicates a classification accuracy of over 90% [60]. Priority is given to entropy in cases where fit indices between the two models were relatively similar. The bootstrapped likelihood ratio test (BLRT) and Vuong-Lo-Mendell-Rubin test (VLMR) were also considered. A significant likelihood ratio test for k classes with *p* < .05 indicates that the specified k-class model improves over a model with k-1 classes [61]. Two hundred random sets of starting values and 50 final stage optimizations were initially used to avoid solutions based on local maxima. Additionally, each latent class was defined with meaningful clinical interpretability [62]. Posterior probabilities from the model were used to assign each participant to their most likely class [63]. One to four latent class models were fitted to determine this study’s optimal number of latent classes.

A logistic regression analysis was then used to identify NSSI-related factors. The statistically significant variables in univariate analyses were incorporated into the logistic model. Statistical significance was evaluated at the 5% level (two-tail test).

## Results

### Overview of the sample

Descriptive statistics for the sample are presented in Table 1. 66.0% of the participants were in junior high school, and 51.2% of the participants lived in rural areas. The vast majority have no siblings (n = 264, 81.0%), no left-behind experience (n = 264, 80.1%) and no family history of mental illness (n = 292, 89.6%).

**Table 1:** Fit indices of the for the LCA models of NSSI, for increasing number of classes (1 to 4)

No. of classes		AIC	BIC	aBIC	BLRT	VLMR	Entropy	N per class
1		5029.824	5079.053	5037.818	—	—	—	—
**2**		**4534.447**	**4636.694**	**4551.051**	***p*** **< .001**	***p*** **< .001**	**0.824**	**129/197**
3		4501.950	4657.213	4527.164	*p* < .001	*p> *0.05	0.840	115/47/164
4		4481.543	4689.822	4515.366	*p* < .001	*p> *0.05	0.749	106/51/119/50

Abbreviations: The values reported in this table are hypothetically derived for illustrative purposes. AIC: Akaike information criterion, BIC: Bayesian information criterion, aBIC: adjust Bayesian information criterion, BLRT: Bootstrap likelihood ratio test, VLMR: Vuong-Lo-Mendell-Rubin test. Bold indicates the selected category.

### Identification of NSSI subgroups

The fit indices of the models generated through LCA are reported in Table 2. The BIC (4657.213) and the entropy (0.840) values both suggested that the three-class solution was possible. Compared with the two-class solution, the three-class solution produced one new subgroup (n = 47) characterized by the lowest NSSI. However, the VLMR of the three and four class solutions were not significant (*p* > .05), indicating a poor latent classification quality. Considering these results, the two-class solution was chosen as the optimal solution. The indices in the diagonal in Appendix 1 shows that the classification accuracy was acceptable, with positive predictive values ranging from 93.4 to 95.9%.

**Table 2:** NSSI characteristics and functions of two subgroups (N = 326)

Variables	Total (N = 326)	Class 1 (n = 129, 39.6%)	Class 2 (n = 197, 60.4%)	*r / d*	*p*
**Clinical characteristics of NSSI**					
Age of onset of NSSI (mean ± SD)	13.0 ± 2.0	12.43 ± 19.6	13.37 ± 2.03	0.5	< 0.001
Suicide attempt (n, %) No Yes	189 (58.0%) 137 (42.0%)	30 (23.3%) 99 (76.7%)	107 (54.3%) 90 (45.7%)	0.29	< 0.001
Length of contemplation before self-injury (n, %) None A few minutes < 1 h > 1 h > 24 h	117(54.3) 80(24.5) 30(9.2) 13(4.0) 26(8.0)	82(63.6) _a_ 28(21.7) 7(5.4) 4(3.1) 8(6.2)	95(48.2) _b_ 52(26.4) 23(11.7) 9(4.6) 18(9.1)	0.16	0.007
Degree of physical pain (n, %) Severe Moderate Mild No	13(4.0) 51(15.6) 162(49.7) 100(30.7)	10(7.8) _a_ 16(12.4) 62(48.1) 41(31.8)	3(1.5) _b_ 35(17.8) 100(50.8) 59(29.9)	0.17	0.027
**NSSI functions**					
Emotion regulation (mean ± SD)	14.07 ± 3.90	16.29 ± 2.87	12.62 ± 3.80	0.45	< 0.001
Attention seeking (mean ± SD)	10.61 ± 4.88	11.54 ± 5.26	10.00 ± 4.53	0.31	< 0.001
Social avoidance (mean, SD)	7.48 ± 3.31	8.47 ± 3.30	6.84 ± 3.16	0.50	< 0.001

Note: class 1: high suicidal ideation NSSI group, class 2: low suicidal ideation NSSI group. a, b: Bonferroni method for multiple comparison, indicates that its column composition ratio is significantly different

**Fig. 2:**
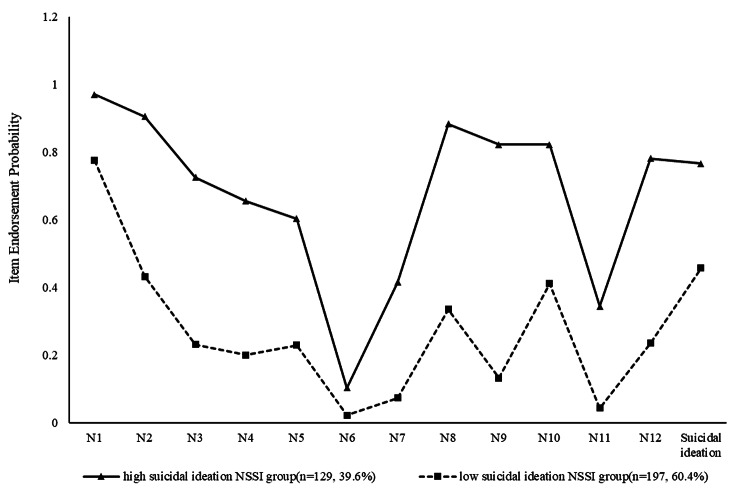
Profiles of latent class of NSSI (N = 326) N1: Cut or carved on your skin; N2 : Hit yourself on purpose; N3: Pulled your hair out; N4: Gave yourself a tattoo; N5: Picked at a wound; N6: Burned your skin; N7: Inserted objects under your nails or skin; N8: Bit yourself; N9: Picked areas of body; N10: Scraped your skin; N11:‘erased’ your skin; N12: Punched walls or objects

A profile was developed based on the conditional probability (see Appendix 2) of a yes for each item. Figure 2 illustrates the profiles of NSSI subtypes for the 2-class model. The y-axis represents the probability of endorsement of specific symptoms, and the x-axis shows indicator variables used for the LCA. Class 1 was labeled as the “high suicidal ideation NSSI group” (n = 129, 39.6%) because this subgroup received a high endorsement of the high frequency of NSSI and suicidal ideation last year. Class 2 (n = 197, 60.4%) was characterized by a low probability of NSSI, a low probability of suicidal ideation and is labeled the “low suicidal ideation NSSI group”.

### Comparison of clinical characteristics of different subgroups

Table 3 shows that the age of onset in the “high suicidal ideation NSSI group” was lower than in the “low suicidal ideation NSSI group”. The “high suicidal ideation NSSI group” had a shorter length of contemplation before NSSI and more physical pain. Of concern is that the probability of SA for one year in the “high suicidal ideation NSSI group” is as high as 76.7%. Regarding NSSI functions yielded, the primary purpose of NSSI is for emotional regulation, followed by attention-seeking and, least of all, social avoidance.

**Table 3:** Comparison of clinical and sociodemographic across subtypes of NSSI (N = 326)

Variables	Total (N = 326)	Class 1 (n = 129, 39.6%)	Class 2 (n = 197, 60.4%)	*r /d*	*p*
Gender (n, %) Girl Boy	263(80.7) 63(19.3)	112(86.8) 17(13.2)	151(76.6) 46(23.4)	0.13	0.023
Age (n, %) 12–15 16–18	219(67.2) 107(32.8)	100(77.5) 29(22.5)	119(60.4) 78(39.6)	0.18	0.001
Grade (n, %) ≤ 9 9–12	215(66.0) 111(34.0)	101(78.3) 28(21.7)	114(57.9) 83(42.1)	0.21	< 0.001
Family location (n, %) Rural City	209(51.2) 199(46.8)	72(52.8) 57(44.2)	100(50.8) 97(49.2)	0.08	0.344
One-child family (n, %) No Yes	264(81.0) 62(19.0)	101(78.3) 28(21.7)	163(82.7) 34(17.3)	0.06	0.317
Left-behind experience (n, %) No Yes	264(81.0) 62(19.0)	95(73.6) 34(26.4)	169(85.8) 28(14.2)	0.15	0.006
Single-parent family (n, %) No Yes	243(74.5) 83(25.5)	87(67.4) 42(32.6)	156(79.2) 41(20.8)	0.13	0.017
Family history of mental illness (n, %) No Yes	292(89.6) 34(10.4)	110(85.3) 19(14.7)	182(92.4) 15(7.6)	0.11	0.040
Depression (mean ± SD)	27.7 ± 6.7	30.5 ± 6.0	26.0 ± 6.6	0.34	< 0.001
Resilience (mean ± SD)	21.9 ± 7.6	20.1 ± 8.2	22.5 ± 7.2	0.20	0.077
Alexithymia (mean ± SD)	70.3 ± 9.7	72.9 ± 9.4	68.6 ± 9.5	0.10	< 0.001
Peer Victimization (mean ± SD)	40.7 ± 18.4	48.8 ± 18.6	35.5 ± 16.3	0.35	< 0.001
Perceived Social Support (mean ± SD)	42.2 ± 17.0	39.7 ± 17.5	43.9 ± 16.5	0.24	0.027

Note: Class 1: high suicidal ideation NSSI group, Class 2: low suicidal ideation NSSI group

### Predictors of latent classes

The logistic regression outcomes (reported in Table 4) indicate that the level of perceived social support is essential, with greater overall levels of perceived social support reducing the chances of belonging to the high suicidal ideation NSSI group (OR = 0.99; 95% CI, 0.97–0.99). Girls, left-behind experience, single-parent family and peer victimization were 6.7 (95%CI: 1.73–26.02), 3.35 (95%CI: 1.08–10.40), 1.84 (95% CI: 1.11–3.04) and 1.04 (95% CI: 1.02–1.05) times more likely to belong to the high suicidal ideation NSSI group. Adolescents who reported depression were 1.10 times more likely (95% CI: 1.05–1.16) to belong to the high suicidal ideation NSSI group.

**Table 4:** Logistic regression for self-injury subtypes (reference class: low suicidal ideation NSSI group)

Variables	High suicidal ideation NSSI group (n = 129, 39.6%)		
	OR	95%CI	*p*
Gender Girl Boy	2.00 Ref	1.09–3.69	0.025
Age 12–15 16–18	Ref 0.85	0.38–1.90	0.686
Grade ≤ 9 > 9	Ref 0.47	0.21–1.05	0.065
Left-behind experience No Yes	Ref 2.08	1.17–3.71	0.013
Single-parent family No Yes	Ref 1.84	1.11–3.04	0.018
Family history of mental illness No Yes	Ref 1.92	0.89–4.13	0.097
Depression	1.10	1.05–1.16	< 0.001
Alexithymia	1.01	0.98–1.05	0.433
Peer Victimization	1.04	1.02–1.05	< 0.001
Perceived Social Support	0.99	0.97–0.99	0.028

Abbreviations: OR: odds ratio, CI: confidence interval; Nagelkerke R^2^ is 0.24

## Discussion

### Two classes of adolescents with NSSI in a clinical sample

In this study, two subgroups of individuals who engage in NSSI were identified using LCA. The two groups were differentiated by NSSI features and severity, demonstrating the heterogeneity of NSSI in a clinical sample of adolescents with depression.

Specifically, the course of NSSI can be classified into two heterogeneous sub-groups or cohorts: the “high suicidal ideation NSSI group” and the “low suicidal ideation NSSI group”, the two classes of adolescents with NSSI identified in this study. The first subgroup identified contains 39.6% of adolescents and is congruent to the “The high-risk NSSI group” and “Multiple functions NSSI/Possible Suicide Ideation group” of earlier studies [64, 65]. Individuals in this group are characterized by high-frequency engagement in NSSI in the past year, participation in more methods of NSSI, and a high probability of a past suicide attempt (compared with class 2). The second group is the “low suicidal ideation NSSI group” consisting of 60.4%. Although this group of participants used various ways to self-injure, they more often used bloodless NSSI methods, such as “pulling hair”, “hitting themselves on purpose”, and “biting their mouths or lips on purpose”, and did not have strong suicidal ideation.

Interestingly, the “high suicidal ideation NSSI group” had the shortest length of contemplation before committing the NSSI. This is inconsistent with data depicting that the severe NSSI group have a more extended period of contemplation before committing NSSI [65]. However, the current study is consistent with Hamza et al. [65], demonstrating that severe adolescents with NSSI have a shorter period of contemplation before committing NSSI, indicating higher negative urgency than normal people. The urgency theory suggests that highly impulsive individuals may be particularly motivated to act rashly in the context of negative emotions because long-term benefits become less important than the immediate short-term gains of emotion regulation [66]. Shorter response latency would be expected to be a behavioral manifestation of trait impulsivity [67]. A shorter latency between urge and injury may also indicate greater severity of NSSI, as measured by frequency and method versatility [68].

In addition, the “high suicidal ideation NSSI group” felt more subjective pain, consistent with previous research [36, 37]. Research has suggested that NSSI frequency, number of NSSI methods, and subjective pain experienced during NSSI were positively associated with a SA history [69]. Perhaps individuals with a history of NSSI have higher pain tolerance and tolerate intense pain for longer than individuals without a history of NSSI [70]. People who reported more subjective pain during NSSI may continuously increase the severity of NSSI to ensure the pain experience, especially when they have become accustomed to the pain caused by the low suicidal ideation NSSI group [37]. It is also possible that people who repeatedly self-injure and report intense subjective pain experiences may show greater pain tolerance rather than a reduction in pain itself [71]. However, Gratz et al. [72] found elevated pain tolerance in the NSSI group, relative to controls, only following a distress manipulation. This phenomenon may indicate that altered pain thresholds in this population may be a transient phenomenon that occurs only during specific periods of high pain, rather than a stable feature [71]. Future research should explore whether pain tolerance is associated with the frequency of NSSI and other painful and adverse events and whether this can, in turn, predict suicidal behavior. Future research could also consider whether the willingness to tolerate pain depends on pain sensitivity, or it may not be an independent structure [69].

### Suicide attempts risk in different subgroups

Joiner concluded that, given the same suicidal ideation (SI), adolescents who self-injured more frequently had higher actual SI than those who self-injured less frequently [73]. The number of NSSI events was significantly and positively correlated with the occurrence and number of SA [74]. NSSI history can predict future suicidal behavior better than SA history [75]. Repeated NSSI may increase the ability of suicide. Specifically, NSSI predisposes one to a greater risk of SA via habituation to the pain and fear needed to carry out suicidal acts [73]. SA with a history of NSSI and SA were more confident in their ability and courage to carry out a lethal SA than those with no history of NSSI [76]. Suicide may gradually become another coping strategy for repeated NSSI.

### Function of NSSI

In both groups of adolescents in this study, NSSI’s most frequently endorsed function was “Emotion regulation”, meaning that NSSI was more to reduce negative emotions or increase positive emotions, consistent with previous findings [77, 78]. According to emotion dysregulation theory, emotion dysregulation may result from a lack of effective emotion regulation skills [79, 80]. When faced with difficulty in regulating emotions, the usual coping mechanisms of the NSSI population may not be sufficient to regulate emotions to reduce the impact of negative emotions. In contrast, NSSI may be a strong enough behavior to serve as a coping mechanism, albeit an inappropriate one [81], and NSSI may be seen as a solution to reducing distress [82]. Emotion regulation corresponds to intrapersonal functions in the “Two-Factor Model” and negative and positive reinforcement functions inherent in the “Four-Factor Model” [83]. This was followed by “Social avoidance” It refers to a self-injured person coping with adversity by avoiding social demands. At the end is “Attention seeking”, inferring the behavior is designed to increase social support and gain the help and attention of others by explaining the self-injured individual. These findings, although preliminary, demonstrate the importance of emotion regulation for NSSI, and as an essential avenue for treatment and prevention.

### Influencing factors of NSSI

This study’s findings are consistent with many other studies, which show that being female is more vulnerable to NSSI [84, 85]. On the one hand, previous studies have found that autoregulatory genes and puberty-related hormonal changes in females could contribute to mood disorders’ pathophysiology, increasing the risk of depression and anxiety in females [86, 87]. On the other hand, the gender-based difference in socio-cultural behavior may also play an important role [88]. For example, females are more likely to be victims of verbal harassment and sexual abuse [89], thus increasing alexithymia and NSSI [90]. In addition, females are more prone to maladaptive emotional regulation strategies [91], which is the core process leading to alexithymia, leading to NSSI and other adverse emotional regulation measures [92].

The findings indicated that teenagers with left behind experiences and being bullied are more likely to participate in severe NSSI. However, peer victimization was not associated with NSSI among non-left-behind children [93]. This result suggests that the relationship between peer victimization and NSSI depends on environmental factors [94]. Compared with the family environment, campus interpersonal relationships may play a more critical role in students’ emotional, cognitive, and personality development [95]. Some studies report that some teenagers use NSSI to regulate interpersonal relationships and control others [96]. Therefore, schools should pay attention to all bullies, especially those students who are long-term victims, and use the necessary means to supervise behavior and prevent bullying, to avoid adverse events.

Young people from single-parent families are also more likely to engage in NSSI. A study that included 59,096 adolescents showed that family structure is an essential factor influencing adolescents’ health behaviors, mental health and perceptions of academic achievement. Adolescents experiencing a shift in family structure may be more vulnerable to health risks than those with intact families [97]. Restructured families are reportedly more prone than other families, especially single-parent families, to face interpersonal problems such as parental conflict and domestic violence and abuse [98]. Understanding these family structure disparities in teenage physical and mental health can help us better understand adolescents and help us create intervention techniques that promote good health.

Clinical studies have shown that the severe NSSI group reported more depressive symptoms, such as lack of pleasure, negative self-evaluation and suicidal ideation [99], which is consistent with the present study’s findings. Studies have found that endorphin levels in the cerebrospinal fluid are lower in patients who self-injure [100]. NSSI can promote the release of endogenous opioid peptides, and the release of opioid peptides not only relieves the sensation of pain caused by NSSI, but also increases pleasure and euphoria and relieves depression, which may explain the recurrent NSSI in depressive individuals [101]. In a survey of 106 adolescents with a history of NSSI, Gordon et al. [102] found that those participants with high-frequency NSSI felt more soothed and relaxed. The emotion regulation function may be one of the reasons for the patients’ repeated NSSI. That is, individuals with depression use NSSI as a form of emotion regulation to alleviate interpersonal difficulties and reduce negative emotions such as low mood, anger, and tension [99].

The current study found that perceived social support is a protective factor against NSSI. Adolescents who have social support are more likely to have improved mental, psychological, and emotional health. The finding concurs with previous findings [103]. Studies have proved that lack of social support has been implicated in the maintenance and severity of NSSI [104], while the perceived presence of support facilitates cessation of the behavior [105]. Such outcomes suggest a protective effect of positive and appropriate social support for NSSI.

### Intervention implications for NSSI

Several interventions appear to hold promise for reducing NSSI, including Dialectical Behavior Therapy for Adolescents (DBT-A) [106], family-centered therapies [107], Mentalization-Based Treatment for adolescents (MBT-A) [108], interpersonal therapy [109], and antipsychotics (e.g. aripiprazole, naltrexone) [110]. LCA is suggestive that different NSSI subgroups may have various treatment indications. Class 1 presents with multiple risk factors and psychopathology. They require training in emotion regulation and distress tolerance skills, building a greater acceptance of uncomfortable emotions [111], reducing impulsivity [112], and training to strengthen interpersonal bonding and family relationships [113]. Considering the risk of suicide, early intensive treatment may be needed during this high-risk period to minimize the risk of suicide behaviors. Class 2 may result from vulnerable adolescent development. One possibility is that people who self-injure with a low frequency and severity can be treated routinely as patients with depression, and NSSI behavior will gradually decrease as depressive symptoms resolve, while those who self-injure more frequently or with a more severe degree of injury, approaching severe NSSI, are in greater need of clinical intervention to prevent the development of a pathological population in the future.

### Limitations

Several limitations should be taken into account in the study: (1) the study required adolescents to recall, over the past year, engagement in NSSI and suicidal behavior so that the study may be subject to recall biases; (2) the study was cross-sectional, precluding the stability of the classes over time. Future studies shedding light on stability and movement across the classes over time, and prediction of treatment outcomes for each class will be valuable; (3) this study found several psychosocial indices that differentiated the two groups. But we cannot be certain about the directionality of effects. Therefore, longitudinal studies are still needed to specifically address whether the psychosocial indicators assessed in this study precede the development of NSSI; (4) LCA is conducted on binary outcomes, and other models (e.g. group-based trajectory model (GBTM)) can be used to validate the classification accuracy further.

## Conclusion

This study has identified two subgroups of NSSI, each subgroup’s clinical characteristics, and the factors associated with each subgroup. The results demonstrated that a higher level of perceived social support is protective against NSSI, while girls, single-parent families, left-behind experience, depression and bullying are risk factors. As an extension of this research, it may be possible to identify adolescents at high risk of NSSI and SA early. To reduce NSSI and promote adolescents’ physical and mental development, more attention should be paid to individuals with risk factors. Schools, families and healthcare providers should focus on adolescents at high risk of NSSI.

## Electronic supplementary material

Below is the link to the electronic supplementary material.

Supplementary Material 1

Supplementary Material 2

## Data Availability

The datasets generated and/or analyzed during the current study are not publicly available due to limitations of ethical approval involving the patient data and anonymity but are available from the corresponding author on reasonable request.

## Abbreviations

AIC: Akaike information criterion
BIC: Bayesian information criterion
aBIC: adjust Bayesian information criterion
BLRT: Bootstrap likelihood ratio test
VLMR: Vuong-Lo-Mendell-Rubin test. Bold indicates the selected category.
