# Supplementary material for: Heterogeneity of non-suicidal self-injury behavior in adolescents with depression: latent class analysis
Source: BMC Psychiatry. 2023 May 1;23:301. doi: 10.1186/s12888-023-04808-7 (PMC10152699; doi:10.1186/s12888-023-04808-7)
Supplement: Supplementary file 1 — Supplementary Material 1 [file 12888_2023_4808_MOESM1_ESM.docx]

# Appendix 1 Most likely latent class membership (row) by latent class (column)

| Model | high suicidal ideation NSSI group | low suicidal ideation NSSI group | |
| --- | --- | --- | --- |
| high suicidal ideation self-injury group | 0.934 | | 0.066 |
| low suicidal ideation self-injury group | 0.041 | | 0.959 |
