# Supplementary material for: Heterogeneity of non-suicidal self-injury behavior in adolescents with depression: latent class analysis
Source: BMC Psychiatry. 2023 May 1;23:301. doi: 10.1186/s12888-023-04808-7 (PMC10152699; doi:10.1186/s12888-023-04808-7)
Supplement: Supplementary file 2 — Supplementary Material 2 [file 12888_2023_4808_MOESM2_ESM.docx]

# Appendix 2 The conditional probability and the latent class probability of the 13 related behaviors variables on the 2 latent classes.

| Variables | Binary | Class 1(n=129, 39.6%) | Class 2 (n=197, 60.4%) |
| --- | --- | --- | --- |
| N1 | 0 | 0.029 | 0.224 |
|  | 1 | 0.971 | 0.776 |
| N2 | 0 | 0.094 | 0.567 |
|  | 1 | 0.906 | 0.433 |
| N3 | 0 | 0.274 | 0.768 |
|  | 1 | 0.726 | 0.232 |
| N4 | 0 | 0.344 | 0.799 |
|  | 1 | 0.656 | 0.201 |
| N5 | 0 | 0.396 | 0.770 |
|  | 1 | 0.604 | 0.230 |
| N6 | 0 | 0.896 | 0.977 |
|  | 1 | 0.104 | 0.023 |
| N7 | 0 | 0.584 | 0.926 |
|  | 1 | 0.416 | 0.074 |
| N8 | 0 | 0.116 | 0.664 |
|  | 1 | 0.884 | 0.336 |
| N9 | 0 | 0.177 | 0.867 |
|  | 1 | 0.823 | 0.133 |
| N10 | 0 | 0.177 | 0.588 |
|  | 1 | 0.823 | 0.412 |
| N11 | 0 | 0.655 | 0.956 |
|  | 1 | 0.345 | 0.044 |
| N12 | 0 | 0.218 | 0.764 |
|  | 1 | 0.782 | 0.236 |
| N13 | 0 | 0.233 | 0.542 |
|  | 1 | 0.767 | 0.458 |
| latent class probability |  | 0.396 | 0.604 |
